# Beyond Pills: Acupressure Impact on Self-Rated Pain and Anxiety Scores

**DOI:** 10.1089/acm.2018.0422

**Published:** 2019-05-09

**Authors:** Elizabeth Monson, Diane Arney, Beth Benham, Rebekah Bird, Erika Elias, Kami Linden, Kimberly McCord, Cathy Miller, Tammy Miller, Lori Ritter, Deanna Waggy

**Affiliations:** ^1^ Heart Failure/Transplant, Intermountain Medical Center, Salt Lake City, Utah.; ^2^ Corvallis, Oregon.; ^3^ Soul Lightening International, New Hampshire.; ^4^ Good Samaritan Regional Medical Center, Corvallis, Oregon.; ^5^ Soul Lightening International, Goldsboro, Maryland.; ^6^ Soul Lightening International, South Bend, Indiana.

**Keywords:** acupressure, pain reduction, anxiety reduction, nurses health, bedside treatment, complementary medicine

## Abstract

***Objectives:*** To determine impact of an acupressure protocol on self-rated pain and anxiety scores.

***Design:*** Retrospective database analysis of self-rated pain and anxiety scores before and immediately after administration of stress release acupressure protocol.

***Participants:*** Participants include hospitalized patients, nurses, and public.

***Intervention:*** Involves a 16-point stress release acupressure protocol.

***Outcome measures:*** Outcome measures involve pre- and post-treatment self-rated pain scores (0–10) with the Wong-Baker Faces Scale and pre- and post-treatment self-rated anxiety scores (0–10) on a visual analog scale.

***Results:*** Five hundred and nineteen acupressure treatments were retrospectively analyzed with pre- and post-treatment self-rated pain and anxiety scores, where 0 represented no pain or anxiety and 10 represented the worst pain and anxiety. Overall, participants demonstrated a two-point decrease in pain scores and a four-point decrease in anxiety scores post-treatment. Hospitalized patients demonstrated a four-point decrease in pain scores and a five-point decrease in anxiety scores post-treatment. Nurses demonstrated a three-point decrease in pain scores and four-point decrease in anxiety scores post-treatment. Public population demonstrated a one-point decrease in pain scores and two-point decrease in anxiety scores post-treatment. Seventy-five percent of participants were highly satisfied with acupressure treatments, and 96% of treatments were administered in less than 30 minutes.

***Conclusions:*** Acupressure is a highly satisfactory complementary therapy that can demonstrate a clinically significant decrease in self-rated pain and anxiety scores.

## Introduction

There is increasing need for treatment options that provide relief from common symptoms such as pain and anxiety, which are efficacious and safe. The health care system is also facing a crisis of pills, opioid, and otherwise, which can increase risk of tolerance, addiction, abuse, and death. Use of effective nonpharmacologic options is now mandated by Joint Commission Guidelines per updated pain management recommendations January 1, 2018.^1^

A recent study compared an opioid versus nonopioid medications for treatment of chronic pain over a 12-month period, and found that using nonopioid therapies offered lower pain scores over time as compared with the opioid therapy group.^2^ Extrapolating from this research, a stronger pain pill is not the most effective way to manage chronic pain. Integrative therapies expand treatment options, thereby offering hope to address symptom management without increased risk of adverse effects.

This study examined the effects of acupressure on self-rated pain and anxiety scores through a retrospective database review. Acupressure is the practice of applying gentle finger pressure to points located along the energy meridians of the body utilized in Traditional Chinese Medicine. Each point exerts certain psychologic, neurologic, and immunologic effects to balance optimum physiologic and psychologic functions. Acupressure is easy to learn and institute in any setting. It is a cost-effective integrative therapy that does not break the protective barrier of the skin, reducing risk of infection and bleeding. Acupressure has been shown to provide efficacious symptom management without significant adverse events.

## Literature Review

Auricular (ear) acupressure has demonstrated the ability to improve heart rate variability, and thus decrease sympathetic nervous system activity.^3^ By decreasing sympathetic nervous system stimulation, the release of stress hormones such as epinephrine and cortisol is decreased, and the relaxation response can be augmented, which may correlate with decreasing levels of pain, stress, and anxiety.^4^

Over the last several decades, there has been a growing interest in acupressure as a therapeutic modality for symptom management in Western health care. Prior randomized-controlled trials (RCTs) have been positive in demonstrating acupressure as an efficacious therapy for symptom treatment of nausea, dysmenorrhea, labor pains, trauma, and dyspnea, improving fatigue and reducing insomnia.^5^

A large military hospital offered an integrative treatment clinic for employees by providing space for 20-min treatments once weekly over the course of 1 year. Three practitioners performed auricular acupuncture, clinical acupressure, or Zero Balancing treatments and tracked ∼2700 individual surveys. Ninety-seven percent of participants felt more relaxed after sessions, and 78 percent noted less pain after treatment with these therapies.^6^

In a study that involved individuals who had just undergone a total knee replacement, auricular acupressure was applied to an intervention group. Both intervention and control groups receive patient-controlled analgesia pumps postoperatively in a postsurgical unit. Intervention group members received auricular acupressure three times per day for 3 days. The auricular acupressure group had a statistically significant (*p* < 0.05) decrease in opioid analgesia and improved passive knee motion as compared with control group.^7^

A systemic review of RCTs using acupressure for control of anxiety among adults was positive with a medium effect size. Among the seven RCTs that met inclusion criteria, all settings were presurgical treatments. Designs for treatment of anxiety were varied, but most involved use of two common acupressure points to treat anxiety. There was an overall statistically significant reduction of anxiety in the acupressure group as compared with sham controls.^8^

## Method, Materials, Participants, and Procedures

Data for this study came from a quality improvement database compiled by Soul Lightening International, a nonprofit organization that focuses on acupressure training and education. Institutional Review Board (IRB) Solutions reviewed and approved the protocols of the research.

Individuals voluntarily elected to receive or self-administer through guided demonstration, an acupressure treatment in a hospital or public setting. The total sample consisted of 519 participants in Indiana, Maryland, New Hampshire, Oregon, Utah, and Wisconsin. Self-reported scores were recorded in the database between 2012 and 2018. Treatments were administered by a nurse, licensed massage therapist, or occupational therapist.

A treatment consisted of a single 16-point acupressure protocol known as the Seva Stress Release Protocol created by Soul Lightening International, see Table 1. Simplified descriptors are used to denote the holds and point locations from Traditional Chinese Medicine. All clinicians received at least 6 h of training on how to administer the protocol.

**Table 1.** Seva Stress Release Acupressure Protocol

1. Leg stretch
2. Middle of back
3. Behind the knees
4. Tops of shoulders
5. Down arms to fingers
6. Neck stretch/brow sweep
7. Below collarbone
8. Armpit and wrist (both sides)
9. Above and below heart
10. Base of rib cage
11. Below kneecaps
12. Behind the knees
13. Hold the toes
14. Neck stretch/brow sweep
15. Root and crown
16. Leg stretch

Seva Stress Release Acupressure Protocol is from Soul Lightening International.

Participants used the Wong-Baker Faces Scale to record or rate their level of pain verbally or through worksheet before and immediately after receiving acupressure treatment, with 0 representing no pain and 10 representing the highest level of pain.^9^

Participants were also invited to self-report their level of anxiety before and immediately after receiving acupressure treatment using a 0–10 visual analog scale, with 0 indicating no anxiety and 10 indicating extreme levels of anxiety.

Hospitalized inpatient participants received treatment from a Seva acupressure-trained registered nurse on a progressive care unit of a 180-bed regional medical center. Pre- and postpain and anxiety scores were reported verbally by the patient immediately before and after acupressure treatment.

Nurse participants received treatments at designated space within 180-bed regional hospital system by a Seva acupressure-trained registered nurse. Participants self-recorded pain and anxiety scores through survey immediately before and after treatment, and submitted written surveys post-treatment.

Public participants self-administered acupressure as guided by a registered nurse, licensed massage therapist, or occupational therapist in a school, workshop, or healing conference setting. Participants self-recorded pain and anxiety scores through survey and submitted written surveys post-treatment.

In addition to reporting post-treatment anxiety and pain scores, all participants gave a rating of satisfaction with 1 indicating the highest level of satisfaction and 10 indicating the highest level of dissatisfaction. The clinician also recorded the total length of the treatment.

## Results

**FIG. 1.**
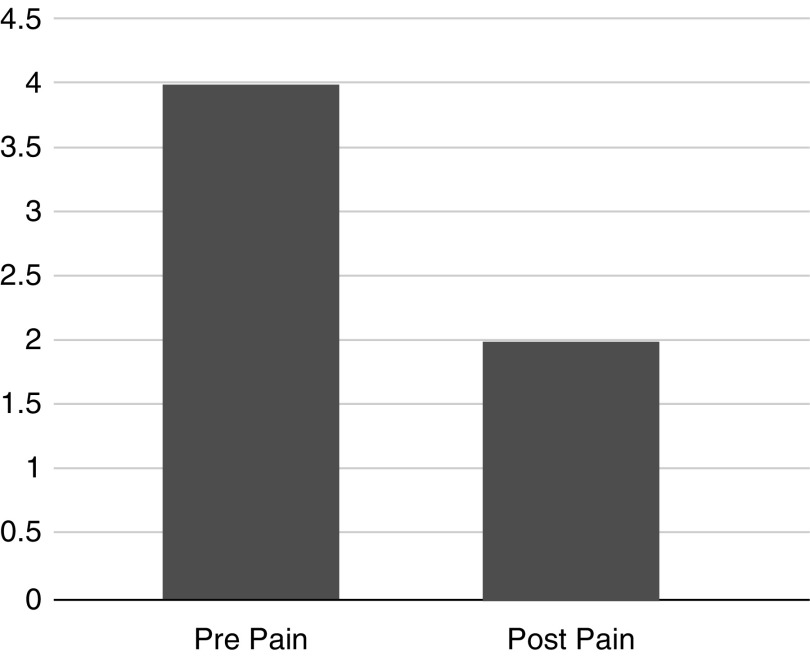
Overall Pain Reduction. Pain score rated on the Wong–Baker Faces score before and after acupressure treatment, 0 representing no pain and 10 representing worst possible pain.

**FIG. 2.**
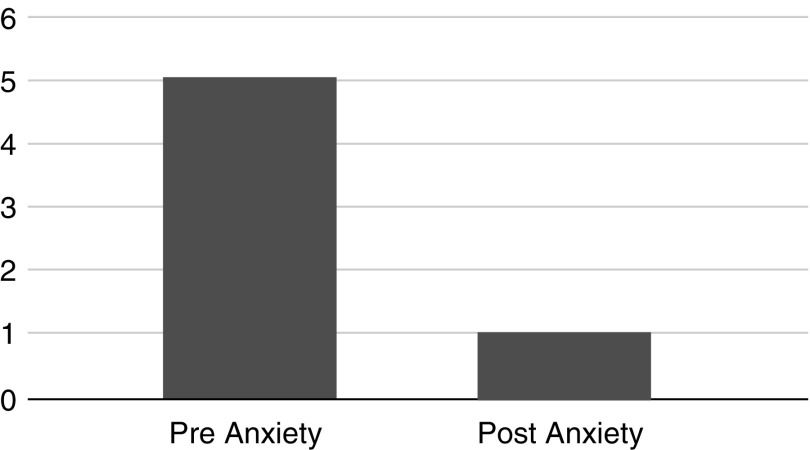
Overall Anxiety Reduction. Anxiety score rated on a 10-point visual analog scale before and after acupressure treatment, 0 representing no anxiety and 10 representing the worst state of anxiety.

Due to the ordinal nature of the data, two Wilcoxon signed-rank tests were conducted to determine the impact of acupressure on anxiety and pain. A Wilcoxon signed-rank test revealed a statistically significant reduction in pain after participation in the acupressure treatment, *z* = −15.81, *p* < 0.05, with a large effect size (*r* = 0.55). The median pain rating decreased from preacupressure (*Md* = 4) to postacupressure (*Md* = 2), see Figure 1. An additional Wilcoxon signed-rank test revealed a statistically significant reduction in anxiety after participation in the acupressure treatment, *z* = −17.231, *p* < 0.05, with a large effect size (*r* = 0.58). The median anxiety rating decreased from preacupressure (*Md* = 5) to postacupressure (*Md* = 1), see Figure 2.

### Hospital population

A Wilcoxon signed-rank test revealed a statistically significant reduction in pain among the hospital population after participation in the acupressure treatment, *z* = −8.7, *p* < 0.05, with a large effect size (*r* = 0.60). The median pain rating decreased from preacupressure (*Md* = 6) to postacupressure (*Md* = 2). In addition, a Wilcoxon signed-rank test revealed a statistically significant reduction in anxiety among the hospital population, *z* = −10.86, *p* < 0.05, with a large effect size (*r* = 0.61). The median anxiety rating decreased from preacupressure (*Md* = 7) to postacupressure (*Md* = 2).

### Nurse population

A Wilcoxon signed-rank test revealed a statistically significant reduction in pain among the nurse population after participation in the acupressure treatment, *z* = −11.5, *p* < 0.05, with a large effect size (*r* = 0.59). The median pain rating decreased from preacupressure (*Md* = 5) to postacupressure (*Md* = 2). In addition, a Wilcoxon signed-rank test revealed a statistically significant reduction in anxiety among the nurse population, *z* = −10.92, *p* < 0.05, with a large effect size (*r* = 0.58). The median anxiety rating decreased from preacupressure (*Md* = 5) to postacupressure (*Md* = 1).

### Public population

Finally, a Wilcoxon signed-rank test revealed a statistically significant reduction in pain among the general population after participation in the acupressure treatment, *z* = −6.6, *p* < 0.05, with a medium effect size (*r* = 0.44). The median pain rating decreased from preacupressure (*Md* = 2) to postacupressure (*Md* = 1). In addition, a Wilcoxon signed-rank test revealed a statistically significant reduction in anxiety among the general population, *z* = −7.68, *p* < 0.05, with a large effect size (*r* = 0.51). The median anxiety rating decreased from preacupressure (*Md* = 3) to postacupressure (*Md* = 1), see Table 2.

**Table 2.** Comparison of Median Scores Before and After Acupressure Treatment Overall and for Specific Populations with Accompanying *z* Scores, *p*-Values, and Effect Size

*Population*	*Prepain (*Md*)*	*Postpain (*Md*)*	z	p	*ES*
Overall	4	2	−15.81	<0.05	*r* = 0.55
Hospital	6	2	−8.7	<0.05	*r* = 0.60
Nurse	5	2	−11.5	<0.05	*r* = 0.59
General	2	1	−6.6	<0.05	*r* = 0.44

*Population*	*Preanxiety (*Md*)*	*Postanxiety (*Md*)*	z	p	*ES*
Overall	5	2	−17.231	<0.05	*r* = 0.58
Hospital	7	2	−10.86	<0.05	*r* = 0.61
Nurse	5	1	−10.92	<0.05	*r* = 0.58
General	3	1	−7.68	<0.05	*r* = 0.51

ES, effect size.

Overall, 96% of participants were able to receive their acupressure treatment in ≤30 min, with 72% of them rating that they were “Most Satisfied” they could be with their treatment.

## Discussion

Overall population of hospitalized inpatients, nurses, and the public demonstrated a 2-point decrease in pain scores and a 4-point decrease in anxiety scores, utilizing the standard 16-point acupressure protocol.

The hospitalized patients represented the population with the highest levels of pain and anxiety. They also demonstrated the largest decrease in symptoms with treatment, with a 4-point average decrease in pain scores and 5-point decrease in anxiety scores. Treatments were performed by registered nurses.

The nurse population demonstrated the next highest pain and anxiety scores. The acupressure treatment demonstrated a 3-point decrease in pain scores and a 4-point decrease in anxiety scores. These treatments were also administered by registered nurses.

The public population had the lowest initial scores for pain and anxiety, and demonstrated a one-point decrease in pain and two-point decrease in anxiety scores. These sessions were guided by Seva acupressure-trained health care professionals and participants self-administered the acupressure therapy.

**FIG. 3.**
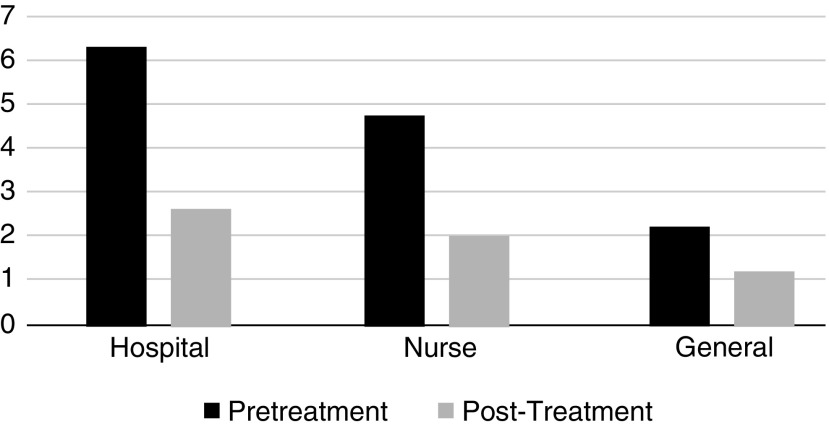
Pain Reduction by Population. Comparison of pain reduction scores among hospitalized patients, nurses, and public.

**FIG. 4.**
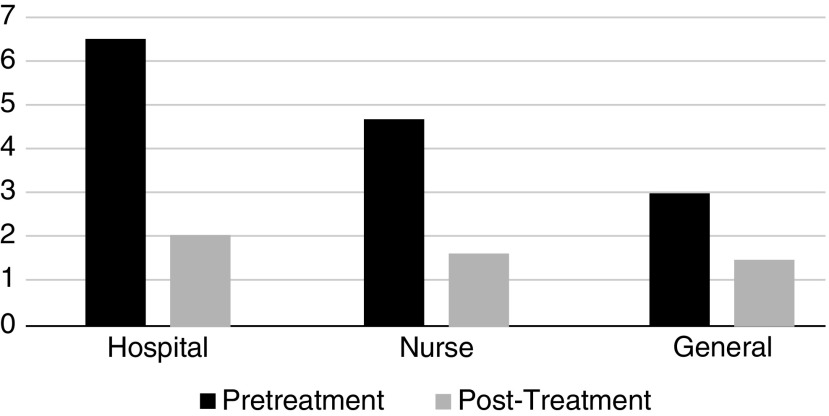
Anxiety Reduction by Population. Comparison of anxiety reduction scores among hospitalized patients, nurses, and public.

In all populations, pain and anxiety scores decreased, a finding that was both statistically and clinically significant (Figs. 3 and 4). There was a greater decrease in pain and anxiety scores when treatments were administered by a registered nurse as compared with the intervention being guided and self-administered by participants. There were no reported adverse events with any of the treatments. Treatments were performed in <30 min, and many were performed in <15 min, equivalent to or less than the onset of action for most pharmacologic agents. Overall, these treatments were rated with the highest level of satisfaction by three quarters of participants.

Present health care systems need additional tools to treat pain and anxiety, which are safe, effective, and avoid the potential for addiction. Health care systems also need cost-effective therapies that can be easily employed in a variety of settings. The health care environment also needs tools that contribute to both patient and provider satisfaction, to improve health and overall wellness outcomes.

Acupressure is an effective tool to address these escalating health care demands. This retrospective analysis has provided evidence that acupressure is a nonpharmacologic tool that can effectively reduce pain and anxiety without adverse events. This intervention also resulted in a high degree of participant satisfaction.

A prospective RCT with standardized tools comparing acupressure with traditional pharmacologic therapy may shed light on impact and efficaciousness of each therapy.

## Limitations

This database analysis is a retrospective review, which did not allow for randomization of participants or control group, thus limiting generalizability of findings. Lack of demographic data other than setting was not captured, which also limits generalizability.

The source of pain and characterization as acute or chronic was not recorded. Rating of anxiety was not based on a standardized tool and utilized a 10-point visual analog scale. Hospitalized patients reported pain and anxiety scores verbally, which could create subject bias. Participants also actively volunteered for treatment, which could create selection bias.

## Conclusion

Retrospective database analysis demonstrates the potential that acupressure can be an effective, complementary nonpharmacologic tool for relief pain and anxiety with a high degree of participant satisfaction.
